# Vital Signs: Disability and Physical Activity — United States, 2009–2012

**Published:** 2014-05-09

**Authors:** Dianna D. Carroll, Elizabeth A. Courtney-Long, Alissa C. Stevens, Michelle L. Sloan, Carolyn Lullo, Susanna N. Visser, Michael H. Fox, Brian S. Armour, Vincent A. Campbell, David R. Brown, Joan M. Dorn

**Affiliations:** 1 Division of Human Development and Disability, National Center on Birth Defects and Developmental Disabilities; 2 Division of Nutrition, Physical Activity, and Obesity, National Center for Chronic Disease Prevention and Health Promotion, CDC

## Abstract

**Background:**

Adults with disabilities are less active and have higher rates of chronic disease than the general population. Given the health benefits of physical activity, understanding physical activity, its relationship with chronic disease, and health professional recommendations for physical activity among young to middle-age adults with disabilities could help increase the effectiveness of health promotion efforts.

**Methods:**

Data from the 2009–2012 National Health Interview Survey (NHIS) were used to estimate the prevalence of, and association between, aerobic physical activity (inactive, insufficiently active, or active) and chronic diseases (heart disease, stroke, diabetes, and cancer) among adults aged 18–64 years by disability status and type (hearing, vision, cognitive, and mobility). The prevalence of, and association between, receiving a health professional recommendation for physical activity and level of aerobic physical activity was assessed using 2010 data.

**Results:**

Overall, 11.6% of U.S. adults aged 18–64 years reported a disability, with estimates for disability type ranging from 1.7% (vision) to 5.8% (mobility). Compared with adults without disabilities, inactivity was more prevalent among adults with any disability (47.1% versus 26.1%) and for adults with each type of disability. Inactive adults with disabilities were 50% more likely to report one or more chronic diseases than those who were physically active. Approximately 44% of adults with disabilities received a recommendation from a health professional for physical activity in the past 12 months.

**Conclusions:**

Almost half of adults with disabilities are physically inactive and are more likely to have a chronic disease. Among adults with disabilities who visited a health professional in the past 12 months, the majority (56%) did not receive a recommendation for physical activity.

**Implications for Public Health:**

These data highlight the need for increased physical activity among persons with disabilities, which might require support across societal sectors, including government and health care.

## Introduction

Persons with disabilities experience limitations in hearing, vision, mobility, or cognition, or have emotional or behavioral disorders. These limitations can negatively impact self-care and activity levels if appropriate accommodations or supports are unavailable (1). The U.S. Census Bureau estimates that approximately 57 million persons of all ages live with a disability (2), and disability-associated health-care expenditures were estimated at nearly $400 billion in 2006 (3). Although disability prevalence increases with age, most adults with disabilities are aged 18–64 years (1). This population is at greater risk for chronic disease and other adverse health outcomes than adults without disabilities (4–6). They are also more likely to see a health-care provider and have a usual source of care compared with those without disabilities (7,8).

Regular aerobic physical activity provides many benefits, including prevention of chronic disease such as coronary heart disease, stroke, type 2 diabetes, and some types of cancer (9,10). The *2008 Physical Activity Guidelines for Americans* (the *2008 Guidelines*) recommend that all adults, including those with disabilities, get ≥150 minutes (2.5 hours) per week of moderate-intensity aerobic physical activity, or ≥75 minutes (1.25 hours) per week of vigorous-intensity aerobic activity, or an equivalent combination (11). Adults with disabilities unable to meet this guideline should regularly engage in physical activity according to their abilities and avoid inactivity (11). However, compared with adults without disabilities, adults with disabilities are more likely to be physically inactive (12–15).

These findings have important implications for promoting physical activity among adults with disabilities. Given the greater risk for chronic disease and higher prevalence of inactivity among persons with disabilities, there is a need to better understand their relationship at younger ages when chronic diseases are typically less prevalent (16) and can be prevented. It is also important to understand if health professionals are recommending physical activity to this subpopulation. This report examines the association between aerobic physical activity and chronic disease for four disability types among adults aged 18–64 years using data from the 2009–2012 National Health Interview Survey (NHIS). Receiving a recommendation from a health professional for physical activity was also assessed using 2010 NHIS data.*

## Methods

NHIS is a continuous, cross-sectional, in-person household survey that is nationally representative of the civilian, noninstitutionalized U.S. population.† The final response rate for the sample adult component ranged from 61%–66% during 2009–2012.

Disability was defined as having serious difficulty in at least one of the following functions: hearing; seeing, even when wearing glasses (vision); concentrating, remembering, or making decisions (cognitive); or walking or climbing stairs (mobility). Based on survey administration in a given year, either the sample adult respondent or the designated household or family member responded to the disability questions. More than one limitation could be reported. Because persons with a mobility limitation might have additional difficulty participating in physical activity, they were only included in the mobility limitation subgroup, even if they reported other limitations. Among persons without a mobility limitation, those with hearing, vision, or cognitive limitations were included in the subgroup for each reported limitation.

Aerobic physical activity levels were defined according to the *2008 Guidelines* using responses to questions on the frequency and duration of leisure-time aerobic physical activity (e.g., walking, bicycling, swimming, and dancing). Minutes of vigorous-intensity activity were multiplied by two when combining with light-intensity to moderate-intensity activities to calculate the moderate intensity-equivalent combination (11). Active (i.e., meeting the aerobic guideline) was defined as participating in ≥150 minutes of moderate-intensity equivalent aerobic activity per week. Insufficiently active was defined as reporting at least one bout of aerobic physical activity per week that lasted ≥10 minutes, but not enough total weekly activity to meet the guideline. Inactive was defined as reporting no bouts of aerobic physical activity per week that lasted ≥10 minutes.

Chronic disease status was determined by respondent report of ever having been told by a doctor or other health professional that he or she had diabetes, cancer, stroke, or heart disease.§ Respondents were categorized as ever having one or more of these chronic diseases, or having none.

Recommendation of physical activity was defined as respondent report of receiving a recommendation from a doctor or other health professional in the past 12 months to begin or continue any type of exercise or physical activity. Analyses using this variable included only sample adult respondents from the 2010 NHIS survey who had seen a doctor or other health professional in the past 12 months.

Disability was assessed for 86,371 sample adult respondents aged 18–64 years. Respondents were excluded if they indicated they were unable to engage in aerobic physical activity (n = 842; 5.4% of adults with disabilities and 0.3% of adults without disabilities), or were missing data for physical activity (n = 1,538), disability status (n = 409), or chronic diseases (n = 115), resulting in an analytic sample of 83,467 adults.

Data were weighted to account for probability of selection and nonresponse, and to adjust for age, sex, and race/ethnicity. The weights were divided by four to account for combining 4 years of data. Prevalence estimates of select demographic and health indicators and receiving a recommendation for physical activity were stratified by disability status and type. Among adults with any disability, prevalence and population estimates of one or more and no chronic diseases were stratified by aerobic physical activity level. Logistic regression was used to calculate adjusted odds ratios (AOR) for the association between physical inactivity and chronic disease for adults with any disability and by disability type, adjusted for sex, age group, race/ethnicity, ratio of family income to poverty threshold, smoking status, and body mass index. Among adults with any disability, prevalence of physical activity levels stratified by receipt of physical activity recommendation, as well as the association between the two, was also estimated.

## Results

Overall, 11.6% of U.S. adults aged 18–64 years, approximately 21.5 million persons, reported a disability. Prevalence estimates by disability type were 1.7% (vision), 2.2% (hearing), 3.0% (cognitive), and 5.8% (mobility). A significantly higher prevalence of adults with disabilities reported having one or more chronic diseases (40.5% versus 13.7%, p<0.001), and being physically inactive (47.1% versus 26.1%, p<0.001) compared with those without disabilities. A significantly higher prevalence of chronic disease and physical inactivity was also noted for each disability type compared with those without a disability (Table 1).

**FIGURE 1:**
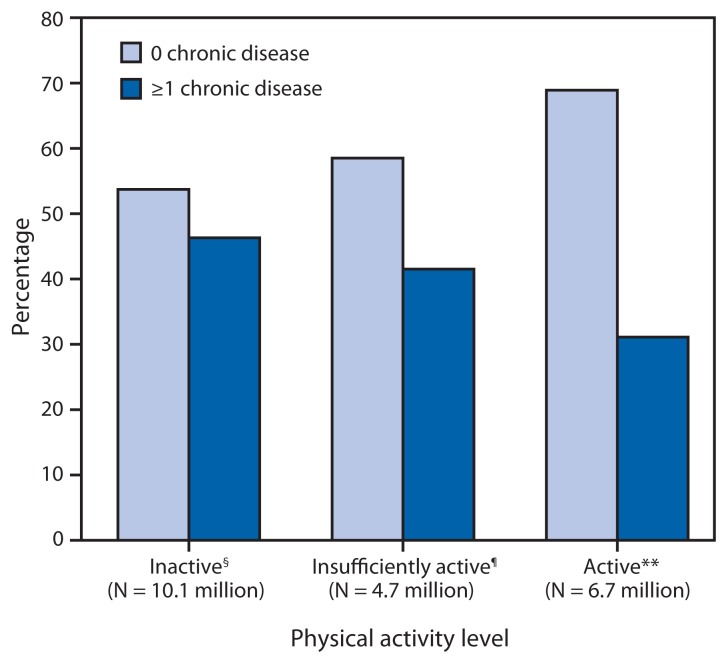
Prevalence and weighted population estimates of the absence or presence of one or more chronic diseases* among adults aged 18–64 years with a disability (N = 10,690), by aerobic physical activity level^†^ — National Health Interview Survey, United States, 2009–2012 * Chronic diseases include diabetes, cancer, stroke, and heart disease. ^†^ Aerobic physical activity levels categorized as active (≥150 minutes/week of moderate-intensity equivalent aerobic activity), insufficiently active (at least one bout of aerobic physical activity per week that lasted ≥10 minutes, but not enough total weekly activity to meet the guideline), or inactive (no bouts of aerobic physical activity per week that lasted ≥10 minutes). ^§^ 0 chronic disease: N = 5.4 million; ≥1 chronic disease: N = 4.7 million. ^¶^ 0 chronic disease: N = 2.8 million; ≥1 chronic disease: N = 2.0 million; weighted population estimates do not add to the overall N of 4.7 million because of rounding. ** 0 chronic disease: N = 4.6 million; ≥1 chronic disease: N = 2.1 million.

Among an estimated 10.1 million inactive adults with disabilities in the United States, 46.3% (approximately 4.7 million adults) reported one or more chronic diseases. Among 6.7 million active adults with disabilities, 31.1% (approximately 2.1 million adults) reported one or more chronic diseases (Figure 1). The prevalence of reporting one or more chronic diseases by disability type among inactive adults was 36.0% (hearing), 36.2% (vision), 34.3% (cognitive), and 54.2% (mobility). The prevalence among active adults was 28.6% (hearing), 26.8% (vision), 24.1% (cognitive), and 42.6% (mobility).

Adults with any disability who were inactive were more likely than those who were active to report one or more chronic diseases (AOR = 1.50; 95% confidence interval [CI] = 1.30–1.72). Significant associations were also found for each disability type except hearing [vision (AOR = 1.52; CI = 1.07–2.14), cognitive (AOR = 1.45; CI = 1.07–1.96), and mobility (AOR = 1.32; CI = 1.09–1.61)] (Table 2).

**FIGURE 2:**
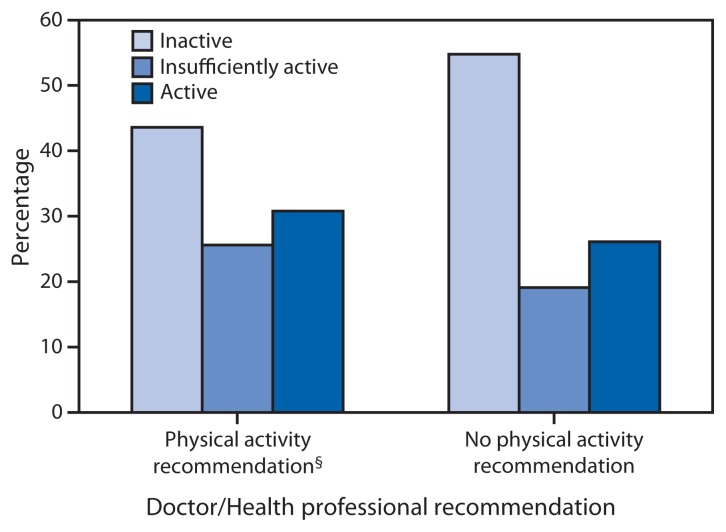
Prevalence* of aerobic physical activity level^†^ among adults aged 18–64 years with a disability (N = 1,090), by whether or not a doctor or health professional recommended exercise or physical activity in the past 12 months — National Health Interview Survey, United States, 2010 * The denominator for this variable also excludes those who have not seen a doctor or other health professional in the past 12 months. ^†^ Aerobic physical activity levels categorized as active (≥150 minutes/week of moderate-intensity equivalent aerobic activity), insufficiently active (at least one bout of aerobic physical activity per week that lasted ≥10 minutes, but not enough total weekly activity to meet the guideline), or inactive (no bouts of aerobic physical activity per week that lasted ≥10 minutes). ^§^ χ^2^ = 5.3, df = 2, p=0.006.

Among adults with a disability who had visited a health professional in the previous 12 months, 44.3% reported that they had received a recommendation for physical activity from a health professional (Table 1). The distribution of aerobic physical activity levels differed significantly by recommendation status (X^2^ = 5.3, df = 2, p=0.006), with a higher prevalence of inactivity among those not receiving a recommendation (54.8% versus 43.6%) (Figure 2). Compared with those who did not report receiving a physical activity recommendation, those who did had significantly higher odds of being active (AOR = 1.82; CI = 1.25–2.64) or insufficiently active (AOR = 1.84; CI = 1.25–2.71) than inactive, even after controlling for demographic characteristics, health behaviors, and the presence of one or more chronic diseases.

### Discussion

Approximately 12% of adults aged 18–64 years reported a disability, and nearly half were inactive. For each disability type, a significantly higher proportion were inactive compared with adults without disabilities; adults with mobility limitations had the highest prevalence of inactivity. Inactive adults with disabilities were 50% more likely to report one or more chronic diseases than adults with disabilities who were active. In 2010, only four in 10 adults with disabilities who visited a health professional in the past 12 months reported receiving a physical activity recommendation. Those who received a recommendation were more likely to be active compared with those who did not receive a recommendation.

Despite recognition of the importance of physical activity promotion among persons with disabilities (4,11,17,18), the prevalence of inactivity remains high, regardless of disability type. A small percentage of adults with disabilities (5.4%) was excluded from this study because they could not engage in physical activity. For other persons with disabilities who could be physically active, barriers exist that limit participation, including 1) limited information about accessible facilities and programs, 2) physical barriers in the built or natural environment, 3) physical or emotional barriers to participating in fitness and recreation activities, and 4) lack of training in accessibility and communication among fitness and recreation professionals (19).

Multisector approaches to improving physical activity are recommended in the *National Prevention Strategy* (18), the *National Physical Activity Plan* (20), and the *2008 Guidelines* (11). Sectors (e.g., government and health care) can each ensure that physical activity promotion efforts include persons with disabilities. CDC currently funds 18 state disability and health programs and five National Public Health Practice and Resource Centers¶ to improve the health and wellness of persons with disabilities. Many of these have developed or used physical activity programs or resources** to address the health needs of persons with disabilities.

The health-care sector is uniquely poised to promote physical activity (21). *Healthy People 2020* objective PA-11 calls for increasing the proportion of physician office visits that include counseling or education related to physical activity.†† This applies to all persons, including those with disabilities. This report shows a positive association between health professional physical activity recommendations and adults with disabilities being physically active. Adults with disabilities are more likely to see a health-care provider and have a usual source of care (7,8), and are encouraged to consult their health-care providers about physical activity appropriate for their abilities (11). These encounters provide multiple opportunities for the health-care sector to promote physical activity among this subpopulation.

The *2008 Guidelines* also apply to persons with disabilities. Doctors and other health professionals can promote physical activity by assessing their patients’ current physical activity levels, emphasizing the importance of physical activity for health, and suggesting online resources and community or local programs suitable for specific abilities.§§ Doctors and health professionals can also review specific resources designed to help them discuss physical activity with patients with disabilities.¶¶

Communities can use strategies recommended in *The Guide to Community Preventive Services*,*** including those that use behavioral and social support approaches, to encourage individual health behavior change and increase physical activity among persons with disabilities.††† Communities can also incorporate environmental and policy approaches such as following Americans with Disabilities Act design guidelines§§§ for fitness centers, worksites, schools, and playgrounds; maintaining safe and accessible parks and trails; and designing sidewalks and streets that are safe and accessible to all persons.¶¶¶ The findings in this report are subject to at least four limitations. First, because of the cross-sectional design of the NHIS, establishing causality or directionality between disability, physical activity, and chronic disease is not possible. Even so, physical activity has the potential to prevent chronic disease and to help manage and improve health for those already having a chronic disease, regardless of directionality. Second, disability estimates are likely conservative because they do not include adults whose disability was considered moderate, those who were unable to engage in aerobic physical activity, and those living in congregate care or institutional settings. However, other datasets using broader definitions of disability show similar disparities in the prevalence of physical activity and inactivity by disability status (12,13,15). Third, the data were either self-reported or provided by a designated household or family member and might be subject to reporting or recall bias. Finally, the NHIS response rates of 61%–66% might have resulted in nonresponse bias.

**Key Points**

- Approximately 21.5 million adults (one in eight) aged 18–64 years have a serious limitation in their hearing, vision, cognition, or mobility.
- Among adults with a disability:
  – Nearly half (approximately 10.1 million) are inactive, meaning they do not get any aerobic physical activity.
  – Those who are inactive are 50% more likely to have a chronic disease than those who get the recommended amount of aerobic physical activity each week.
  – Approximately 5.4 million inactive adults with disabilities who do not currently have diabetes, stroke, heart disease or cancer, are missing opportunities to protect against these chronic diseases through physical activity.
  – Approximately 4.7 million inactive adults with disabilities who already have chronic disease are missing opportunities to manage or mitigate the effects of these diseases.
  – Approximately 44% of adults who saw a doctor or other health professional in the past 12 months received a physical activity recommendation and were more likely to be active than those who did not receive a recommendation.
- Doctors and health professionals can promote physical activity among their patients with disabilities.
- Additional information is available at http://www.cdc.gov/vitalsigns.

Approximately half of adults aged 18–64 years with disabilities (approximately 10.1 million adults) are missing the opportunity to protect or improve their health and potentially delay or prevent chronic disease onset through physical activity. Providing safe, appropriate, and accessible physical activity options to persons with disabilities requires support across sectors, including health-care, to help persons with disabilities more easily engage in this essential health behavior.

## Figures and Tables

**TABLE 1:** Prevalence of selected demographic characteristics and health behaviors among adults aged 18–64 years (N = 83,467), by disability type* — National Health Interview Survey, United States, 2009–2012

	No mobility limitation											
				
	Hearing		Vision		Cognitive		Mobility limitation		Any disability		No disability	
						
Characteristic	%	(95% CI)	%	(95% CI)	%	(95% CI)	%	(95% CI)	%	(95% CI)	%	(95% CI)
**Total**	**2.2**	**(2.0–2.3)**	**1.7**	**(1.6–1.9)**	**3.0**	**(2.9–3.2)**	**5.8**	**(5.6–6.1)**	**11.6**	**(11.3–11.9)**	**88.4**	**(88.1–88.7)**
**Sex**												
Male	64.1	(61.2–66.8)	48.9	(45.6–52.2)	50.9	(48.3–53.5)	43.5	(41.7–45.4)	49.1	(47.8–50.4)	49.3	(48.8–49.8)
Female	35.9	(33.2–38.8)	51.1	(47.8–54.4)	49.1	(46.5–51.7)	56.5	(54.6–58.3)	50.9	(49.6–52.2)	50.7	(50.2–51.2)
**Age group (yrs)**												
18–44	33.7	(30.9–36.7)	47.4	(43.8–51.0)	60.9	(58.3–63.5)	25.2	(23.6–26.8)	37.6	(36.3–38.9)	60.9	(60.4–61.5)
45–64	66.3	(63.3–69.1)	52.6	(49.0–56.2)	39.1	(36.5–41.7)	74.8	(73.2–76.4)	62.4	(61.1–63.7)	39.1	(38.5–39.6)
**Race/Ethnicity**												
White	75.2	(72.6–77.7)	59.3	(55.9–62.7)	64.9	(62.4–67.4)	64.4	(62.7–66.0)	65.9	(64.7–67.1)	65.0	(64.5–65.6)
Black	7.6	(6.2–9.4)	14.4	(12.3–16.7)	16.0	(14.1–18.0)	18.1	(16.8–19.4)	15.4	(14.5–16.4)	11.6	(11.3–12.0)
Hispanic	12.0	(10.2–13.9)	19.6	(17.0–22.5)	13.5	(11.9–15.4)	12.5	(11.5–13.7)	13.4	(12.6–14.3)	16.1	(15.6–16.5)
Other†	5.3	(4.1–6.6)	6.7	(5.3–8.6)	5.6	(4.7–6.7)	5.1	(4.4–5.9)	5.3	(4.7–5.8)	7.3	(7.0–7.6)
**Family income to poverty threshold (ratio)**												
<1.0	15.3	(13.2–17.6)	25.6	(22.8–28.7)	33.5	(30.8–36.2)	30.5	(28.8–32.2)	27.7	(26.5–29.0)	12.5	(12.2–12.9)
1.0 to < 2.0	16.1	(14.0–18.5)	23.8	(20.7–27.1)	25.3	(22.9–27.9)	25.0	(23.4–26.6)	23.4	(22.2–24.6)	15.5	(15.2–15.9)
≥2.0	68.7	(65.7–71.5)	50.6	(47.1–54.2)	41.2	(38.4–44.2)	44.6	(42.6–46.5)	48.9	(47.4–50.4)	71.9	(71.4–72.5)
**Smoking status**												
Current smoker	29.6	(26.8–32.6)	35.1	(31.8–38.4)	39.1	(36.5–41.7)	33.1	(31.4–34.8)	33.6	(32.4–34.9)	19.6	(19.2–20.0)
Former smoker	24.2	(21.7–26.9)	17.9	(15.5–20.7)	15.8	(14.0–17.8)	25.0	(23.5–26.6)	22.2	(21.2–23.3)	17.4	(17.1–17.8)
Never smoker	46.2	(43.2–49.1)	47.0	(43.7–50.3)	45.1	(42.4–47.8)	41.9	(40.1–43.7)	44.2	(42.9–45.5)	63.0	(62.5–63.5)
**Body mass index**												
Obese	34.5	(31.5–37.5)	39.0	(35.6–42.4)	34.8	(32.1–37.6)	54.5	(52.8–56.3)	45.0	(43.8–46.3)	28.6	(28.2–29.1)
Overweight	37.4	(34.2–40.7)	28.8	(25.9–31.8)	30.0	(27.5–32.6)	26.1	(24.5–27.7)	29.1	(27.9–30.2)	34.0	(33.5–34.5)
Normal or underweight	28.2	(25.4–31.1)	32.3	(29.3–35.4)	35.2	(32.6–37.9)	19.4	(18.0–20.9)	25.9	(24.8–27.1)	37.4	(36.9–37.9)
**Chronic disease**												
0 of 4	68.0	(65.0–70.8)§	67.2	(64.0–70.3)§	70.9	(68.4–73.3)§	48.6	(46.7–50.4)§	59.5	(58.2–60.8)§	86.3	(85.9–86.6)
≥1 of 4	32.0	(29.2–35.0)§	32.8	(29.7–36.0)§	29.1	(26.7–31.6)§	51.4	(49.6–53.3)§	40.5	(39.2–41.8)§	13.7	(13.4–14.1)
Diabetes	10.8	(9.0–12.8)	14.8	(12.5–17.4)	10.1	(8.6–12.0)	27.0	(25.4–28.6)	19.0	(18.1–20.0)	5.0	(4.8–5.2)
Cancer	10.2	(8.5–12.2)	8.0	(6.3–10.1)	8.2	(6.9–9.8)	11.5	(10.5–12.6)	10.0	(9.2–10.8)	4.3	(4.1–4.5)
Stroke	3.6	(2.5–5.1)	5.3	(3.9–7.1)	5.0	(4.0–6.3)	9.4	(8.4–10.4)	6.6	(6.1–7.3)	0.7	(0.7–0.8)
Heart disease	16.9	(14.7–19.3)	15.4	(13.1–18.1)	14.5	(12.6–16.6)	27.5	(25.9–29.1)	20.9	(19.9–22.0)	5.7	(5.5–6.0)
**Aerobic physical activity** ¶												
Active	45.2	(42.2–48.2)§	40.9	(37.7–44.2)§	38.3	(35.6–41.1)§	20.6	(19.2–22.1)§	31.0	(29.7–32.2)§	53.7	(53.1–54.2)
Insufficiently active	21.7	(19.2–24.5)	22.7	(20.1–25.6)	21.6	(19.4–23.8)	22.0	(20.6–23.5)	21.9	(20.9–23.0)	20.2	(19.8–20.6)
Inactive	33.1	(30.3–36.1)§	36.4	(33.4–39.5)§	40.1	(37.5–42.9)§	57.4	(55.6–59.1)§	47.1	(45.8–48.5)§	26.1	(25.6–26.7)
**Physical activity recommendation** **												
Yes	43.7	(34.4–53.4)	40.6	(31.2–50.9)	42.7	(35.1–50.6)	46.4	(41.2–51.6)	44.3	(40.4–48.2)	31.0	(29.5–32.4)

**Abbreviation:** CI = confidence interval.

* Respondents could report more than one limitation and were included in the analysis for each reported limitation. The only exception was that those with a mobility limitation, regardless of any additional limitations, were only included in the mobility limitation subgroup.

† Other includes American Indian/Alaska Native, Asian, multiple race, and “race group not releasable.”

§ p-value <0.001 when compared with no disability. Comparisons only made for aerobic physical activity and chronic disease.

¶ Aerobic physical activity levels were categorized as active (≥150 minutes/week of moderate-intensity equivalent aerobic activity), insufficiently active (at least one bout of aerobic physical activity per week that lasted ≥10 minutes, but not enough total weekly activity to meet the guideline), or inactive (no bouts of aerobic physical activity per week that lasted at least 10 minutes).

** Data from 2010 only; the denominator for this variable also excludes those who have not seen a doctor or other health professional in the past 12 months.

**TABLE 2:** Adjusted odds ratios* for reporting at least one of four chronic diseases† among adults aged 18–64 years (N = 10,690), by disability type§ — National Health Interview Survey, United States, 2009–2012

	No mobility limitation									
			
	Hearing		Vision		Cognitive		Mobility limitation		Any disability	
					
Characterisitic	AOR	(95% CI)	AOR	(95% CI)	AOR	(95% CI)	AOR	(95% CI)	AOR	(95% CI)
**Sex**										
Male	1.16	(0.85–1.59)	1.31	(0.95–1.82)	0.90	(0.69–1.17)	0.99	(0.85–1.17)	0.95	(0.84–1.07)
Female	Ref	Ref	Ref	Ref	Ref	Ref	Ref	Ref	Ref	Ref
**Age group (yrs)**										
18–44	Ref	Ref	Ref	Ref	Ref	Ref	Ref	Ref	Ref	Ref
45–64	2.87	(2.02–4.09)	2.68	(1.94–3.71)	3.18	(2.44–4.14)	3.20	(2.67–3.85)	3.32	(2.91–3.78)
**Race/Ethnicity**										
White	Ref	Ref	Ref	Ref	Ref	Ref	Ref	Ref	Ref	Ref
Black	0.92	(0.58–1.47)	1.07	(0.69–1.65)	0.76	(0.54–1.07)	1.08	(0.90–1.30)	1.02	(0.88–1.18)
Hispanic	0.71	(0.44–1.14)	0.97	(0.63–1.50)	0.86	(0.59–1.23)	1.02	(0.81–1.27)	0.95	(0.81–1.12)
Other¶	1.48	(0.83–2.61)	1.40	(0.82–2.39)	1.21	(0.74–1.99)	1.31	(0.93–1.85)	1.31	(1.03–1.67)
**Family income to poverty threshold (ratio)**										
<1.0	2.42	(1.62–3.60)	1.24	(0.83–1.86)	1.20	(0.88–1.65)	1.40	(1.16–1.68)	1.49	(1.30–1.71)
1.0 to <2.0	1.56	(1.03–2.37)	1.29	(0.87–1.91)	0.90	(0.65–1.23)	1.55	(1.27–1.88)	1.47	(1.27–1.69)
≥2.0	Ref	Ref	Ref	Ref	Ref	Ref	Ref	Ref	Ref	Ref
**Smoking status**										
Current smoker	1.29	(0.92–1.82)	1.17	(0.83–1.64)	1.13	(0.84–1.51)	0.97	(0.81–1.17)	1.08	(0.95–1.24)
Former smoker	1.48	(1.04–2.10)	1.70	(1.13–2.56)	1.67	(1.18–2.36)	1.25	(1.02–1.52)	1.41	(1.22–1.63)
Never smoker	Ref	Ref	Ref	Ref	Ref	Ref	Ref	Ref	Ref	Ref
**Body mass index**										
Obese	1.48	(1.02–2.16)	1.41	(0.96–2.08)	1.19	(0.87–1.64)	1.62	(1.31–2.00)	1.64	(1.42–1.90)
Overweight	1.17	(0.80–1.70)	1.18	(0.78–1.79)	0.95	(0.69–1.31)	1.16	(0.92–1.46)	1.13	(0.96–1.33)
Normal or underweight	Ref	Ref	Ref	Ref	Ref	Ref	Ref	Ref	Ref	Ref
**Aerobic physical activity** **										
Active	Ref	Ref	Ref	Ref	Ref	Ref	Ref	Ref	Ref	Ref
Insufficiently active	1.18	(0.79–1.76)	1.63	(1.08–2.45)	1.12	(0.80–1.57)	1.28	(1.00–1.62)	1.34	(1.13–1.58)
Inactive	1.25	(0.89–1.74)	1.52	(1.07–2.14)	1.45	(1.07–1.96)	1.32	(1.09–1.61)	1.50	(1.30–1.72)

**Abbreviations:** AOR = adjusted odds ratio; CI = confidence interval; Ref = referent.

* Odds ratios adjusted for sex, age, race/ethnicity, ratio of family income to poverty threshold, smoking status, and body mass index.

† Chronic diseases include diabetes, cancer, stroke, and heart disease.

§ Respondents could report more than one limitation and were included in the analysis for each reported limitation. The only exception was that those with a mobility limitation, regardless of any additional limitations, were only included in the mobility limitation subgroup.

¶ Other includes American Indian/Alaska Native, Asian, multiple race, and “race group not releasable.”

** Aerobic physical activity levels categorized as active (≥150 minutes/week of moderate-intensity equivalent aerobic activity), insufficiently active (at least one bout of aerobic physical activity per week that lasted ≥10 minutes, but not enough total weekly activity to meet the guideline), or inactive (no bouts of aerobic physical activity per week that lasted ≥10 minutes).

## Footnotes

* Between 2009 and 2012, the NHIS question assessing doctor or health professional recommendation of physical activity was only asked in 2010.

† Additional information available at http://www.cdc.gov/nchs/nhis.htm.

§ Heart disease was defined as coronary heart disease, angina, heart attack, myocardial infarction, or any other heart condition or heart disease.

¶ Additional information available at http://www.cdc.gov/ncbddd/disabilityandhealth/partners-and-programs.html.

** Examples include Health Matters (http://www.rrtcadd.org/blog/files/f964400825250eb9a0460f04d62d957d-45.html), Living Well with a Disability (http://livingandworkingwell.org), Steps to Your Health (http://sciodh.com/materials), Fit for Life (http://www.health.ny.gov/publications/1228.pdf), 14 Weeks to a Healthier You Program (http://www.nchpad.org/14weeks), Health Meet (http://www.thearc.org/page.aspx?pid=3686), Healthy Athletes (http://www.specialolympics.org/healthy_athletes.aspx), Limb Loss Resource Center exercise resources (http://www.amputee-coalition.org/limb-loss-resource-center/resources-by-topic/exercise/index.html), Paralysis Resource Center fact sheet on fitness and exercise (http://www.christopherreeve.org/atf/cf/%7b173bca02-3665-49ab-9378-be009c58a5d3%7d/fitnessexercise6-13.pdf), and EnhanceFitness (http://www.projectenhance.org/enhancefitness.aspx).

†† Additional information available at http://www.healthypeople.gov/2020/topicsobjectives2020/objectiveslist.aspx?topicId=33.

§§ Examples can be found at CDC’s Disability and Physical Activity website (http://www.cdc.gov/disabilities/pa), Be Active Your Way: A Guide for Adults (http://www.health.gov/paguidelines/pdf/adultguide.pdf), Walk with Ease (http://www.arthritis.org/resources/community-programs/walk-with-ease/), and Go4life (http://go4life.nia.nih.gov/).

¶¶ Sample resources are available online at http://www.cdc.gov/ncbddd/disabilityandhealth/pdf/disabilityposter_photos.pdf, http://exerciseismedicine.org, http://www.ahrq.gov/professionals/quality-patient-safety/quality-resources/tools/literacy-toolkit/index.html, http://certification.acsm.org/acsm-inclusive-fitness-trainer, and http://www.cdc.gov/healthliteracy/developmaterials/plainlanguage.html, and in print (Durstine JL, Moore GE, Painter PL, Roberts SO, eds. ACSM’s exercise management for persons with chronic diseases and disabilities. Champaign, IL: American College of Sports Medicine; 2009).

*** Available at http://www.thecommunityguide.org/pa/index.html.

††† Examples include Health Matters (http://www.rrtcadd.org/blog/files/f964400825250eb9a0460f04d62d957d-45.html), Living Well with a Disability (http://livingandworkingwell.org/), and Steps to your Health (http://sciodh.com/materials).

§§§ Available at http://www.access-board.gov/guidelines-and-standards/buildings-and-sites/about-the-ada-standards/ada-standards.

¶¶¶ Examples include using curb cuts to reduce barriers to walking or wheeling and audible traffic signals for persons with vision impairments.
